# Supplementary material for: Acute Kidney Injury in Pediatric Patients on Extracorporeal Membrane Oxygenation: A Systematic Review and Meta-analysis
Source: Medicines (Basel). 2019 Nov 1;6(4):109. doi: 10.3390/medicines6040109 (PMC6963279; doi:10.3390/medicines6040109)
Supplement: Supplementary file 1 [file medicines-06-00109-s001.pdf]

Supplementary Materials: Acute Kidney Injury in Pediatric Patients on Extracorporeal Membrane Oxygenation: A Systematic Review and Meta-analysis

Panupong Hansrivijit, Ploypin Lertjitbanjong, Charat Thongprayoon, Wisit Cheungpasitporn, Narothama Reddy Aeddula, Sohail Abdul Salim,Api Chewcharat, Kanramon Watthanasuntorn, Narat Srivali, Michael A. Mao, Patompong Ungprasert, Karn Wijarnpreecha, Wisit Kaewput and Tarun Bathini

Data S1: Search terms for systematic review.

Database: Ovid MEDLINE (783 articles)

1. exp acute kidney injury/
2. acute kidney injury$.mp
3. exp acute renal failure/
4. acute renal failure$.mp.
5. exp renal insufficiency/
6. renal insufficiency$.mp.
7. exp dialysis/
8. dialysis$.mp.
9. hemodialysis$.mp.
10. renal replacement therapy$.mp.
11. hemofiltration$.mp.
12. hemodiafiltration$.mp.
13. 1 or 2 or 3 or 4 or 5 or 6 or 7 or 8 or 9 or 10 or 11 or 12
14. Extracorporeal Membrane Oxygenation.mp
15. Exp Extracorporeal Membrane Oxygenation/
16. ECMO.mp
17. 14 or 15 or 16
18. 13 and 17

Database: EMBASE (952 articles)
('extracorporeal oxygenation' OR 'extracorporeal membrane oxygenation device' OR 'extracorporeal membrane oxygenation cannula') AND 'acute kidney failure'
Cochrane Database (6 articles)

"extracorporeal membrane oxygenation" AND "acute kidney injury"

Study name	Event rate	Lower limit	Upper limit	Z-Value	p-Value
	Statistics for each study				
Gadepalli et al	0.706	0.588	0.802	3.289	0.001
Zwiers et al	0.632	0.570	0.691	4.064	0.000
Fleming et al	0.603	0.570	0.636	5.920	0.000
Elella et al	0.898	0.792	0.954	5.058	0.000
	0.692	0.597	0.773	3.806	0.000

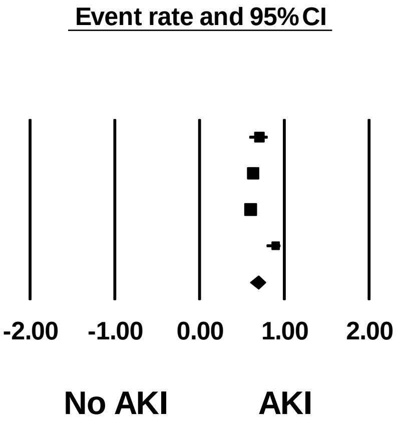

Figure S1. Forest plots of the included studies assessing incidence of AKI limited to studies with standard AKI definitions was performed and demonstrated the pooled estimated incidence of AKI.

Group by Cardiac surgery	Study name	Event rate	Lower limit	Upper limit	Z-Value	p-Value
		Statistics for each study				
No	Gadepalli et al	0.706	0.588	0.802	3.289	0.00100
No	Hoffman	0.500	0.225	0.775	0.000	1.00000
No	Zwiers et al	0.632	0.570	0.691	4.064	0.00005
No	Fleming et al	0.603	0.570	0.636	5.920	0.00000
No	Askenazi et al	0.205	0.188	0.223	-24.243	0.00000
No		0.523	0.273	0.762	0.168	0.86623
Yes	Elella et al	0.898	0.792	0.954	5.058	0.00000
Yes	Borasino et al	0.700	0.560	0.810	2.746	0.00604
Yes		0.814	0.544	0.942	2.225	0.02609

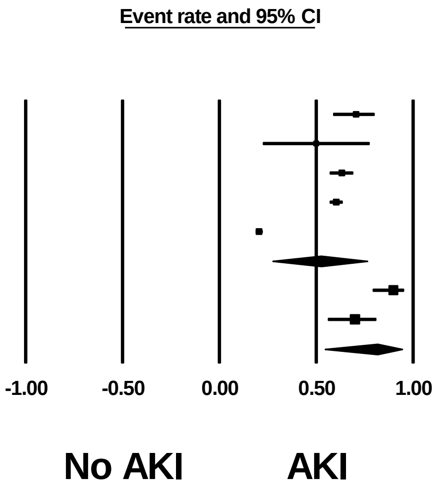

Figure S2. Forest plots of the included studies assessing incidence of AKI based on cardiac surgery status.

Group by Cardiac surgery	Study name	Event rate	Lower limit	Upper limit	Z-Value	p-Value	Relative weight
		Statistics for each study					
No	Hoover et al	0.302	0.215	0.407	-3.562	0.00037	25.26
No	Gadepalli et al	0.162	0.092	0.269	-4.996	0.00000	22.36
No	Goto et al	0.500	0.260	0.740	0.000	1.00000	16.19
No	Askenazi et al	0.428	0.406	0.450	-6.344	0.00000	28.99
No	Yang et al	0.083	0.012	0.413	-2.296	0.02169	7.19
No		0.302	0.187	0.449	-2.590	0.00960	
Yes	Kolovos et al	0.351	0.252	0.466	-2.518	0.01181	22.15
Yes	Gbadegesin et al	0.404	0.314	0.501	-1.949	0.05132	22.72
Yes	Ricci et al	0.300	0.100	0.624	-1.228	0.21950	14.47
Yes	Elella et al	0.898	0.792	0.954	5.058	0.00000	19.08
Yes	Borasino et al	0.520	0.383	0.654	0.283	0.77736	21.58
Yes		0.522	0.325	0.712	0.207	0.83628	

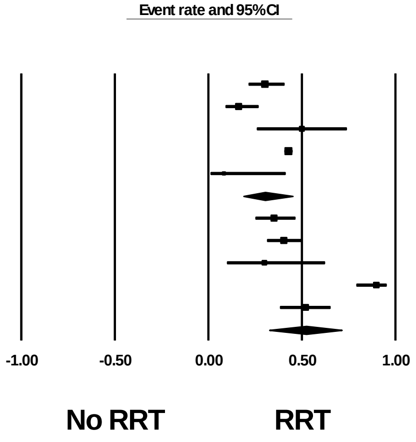

Figure S3. Forest plots of the included studies assessing incidence of severe AKI requiring RRT based on cardiac surgery status.

Study name	Statistics for each study					Relative weight
	Event rate	Lower limit	Upper limit	Z-Value	p-Value	
Askenazi et al	0.657	0.609	0.702	6.175	0.000	34.43
Zwiers et al	0.281	0.216	0.357	-5.223	0.000	33.62
Elella et al	0.623	0.486	0.742	1.767	0.077	31.95
	0.517	0.265	0.760	0.122	0.903	

Event rate and 95% CI

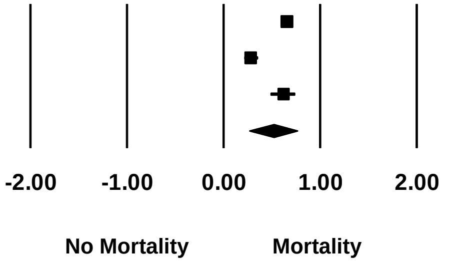

Figure S4. Forest plots of the included studies assessing hospital and/or 90-day mortality rates of pediatric patients on ECMO with AKI.

Study name	Statistics for each study					Relative weight
	Event rate	Lower limit	Upper limit	Z-Value	p-Value	
Kolovos et al	0.769	0.572	0.892	2.587	0.010	12.84
Hoover et al	0.269	0.134	0.467	-2.258	0.024	13.33
Gbadegesin et al	0.833	0.690	0.918	3.887	0.000	13.94
Gadepalli et al	0.727	0.553	0.852	2.509	0.012	14.44
Goto et al	0.286	0.072	0.673	-1.095	0.273	6.98
Askenazi et al	0.580	0.546	0.613	4.604	0.000	20.32
Ricci et al	0.667	0.154	0.957	0.566	0.571	3.98
Borasino et al	0.615	0.421	0.779	1.166	0.244	14.17
	0.618	0.488	0.734	1.777	0.076	

Event rate and 95% CI

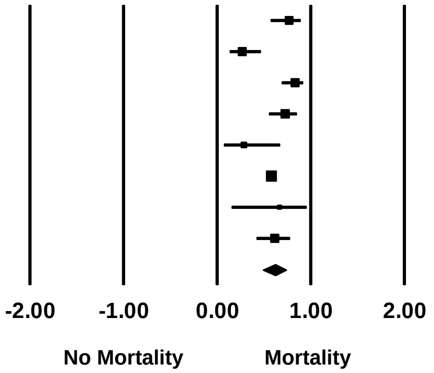

Figure S5. Forest plots of the included studies assessing hospital and/or 90-day mortality rates of pediatric patients on ECMO with severe AKI requiring RRT .

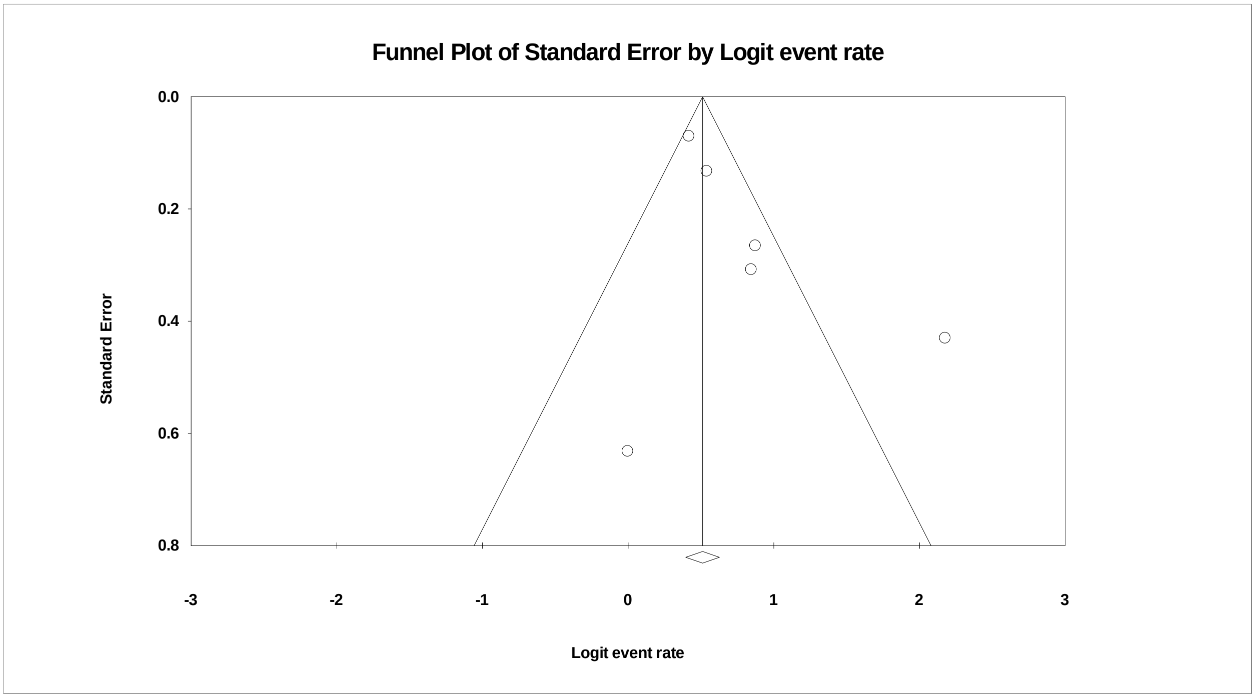

Figure S6. Funnel plot evaluating for publication bias evaluating incidence of AKI while on ECMO.

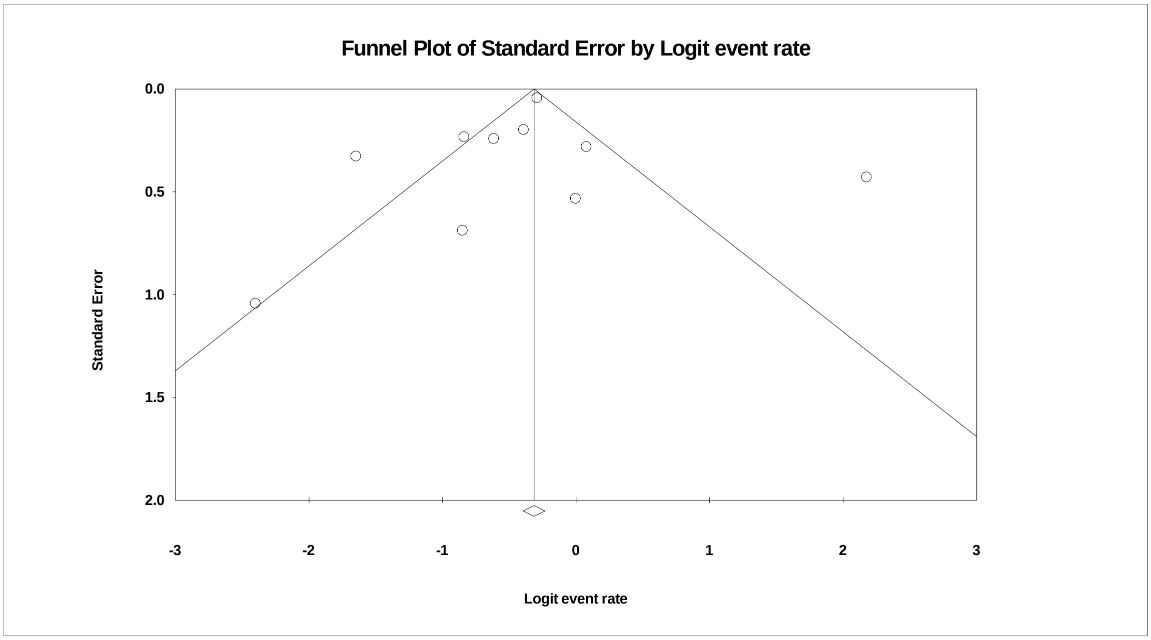

Figure S7: Funnel plot evaluating for publication bias evaluating incidence of severe AKI requiring RRT while on ECMO.
